# Supplementary material for: Geospatial analysis of toponyms in geotagged social media posts
Source: PLoS One. 2025 Jun 5;20(6):e0325022. doi: 10.1371/journal.pone.0325022 (PMC12140283; doi:10.1371/journal.pone.0325022)
Supplement: S1 Appendix — (PDF) [file pone.0325022.s001.pdf]

# Supporting Information for "Geospatial analysis of toponyms in geotagged social media posts"

Takayuki Hiraoka[1], Takashi Kirimura[2], Naoya Fujiwara[3,4,5,6]

**1** Department of Computer Science, Aalto University, Espoo, Finland
**2** Department of Kyoto Studies, Kyoto Sangyo Univerisity, Kyoto, Japan
**3** Graduate School of Information Sciences, Tohoku University, Sendai, Japan
**4** PRESTO, Japan Science and Technology Agency, Kawaguchi, Japan
**5** Institute of Industrial Science, The University of Tokyo, Tokyo, Japan
**6** Center for Spatial Information Science, The University of Tokyo, Kashiwa, Japan

* takayuki.hiraoka@aalto.fi

## 1 Definition of relative entropy

In the main text, we use relative entropy (Kullback–Leibler (KL) divergence) to measure the dissimilarity between the empirical and model distributions. Here, we describe the definition of relative entropy in detail.

Each distribution is defined in the two-dimensional space of the total number of posts per cell, denoted by $n_{\mathrm{all}}$, and the number of posts with word $w$, denoted by $n_w$. Specifically, we only consider the values of $n_{\mathrm{all}}$ that are empirically observed, and the values of $n_w$ equal to or smaller than $n_{\mathrm{all}}$. That is, the support of each distribution is given by

$$\{(n_{\mathrm{all}}, n_w) \in \mathbb{N} \times \mathbb{N} \mid (\exists c \in \Gamma)[n_{\mathrm{all}} = n_{\mathrm{all},c}], n_w \leq n_{\mathrm{all}}\}$$

for a set of grid cells $\Gamma$. Given a set of empirical numbers of posts $\{n_{w,c}\}$ containing word $w$ for $c \in \Gamma$, relative entropy is defined as

$$D_{\mathrm{KL}}(Q_w \parallel P_w) = \frac{1}{|\Gamma|} \sum_{c \in \Gamma} \left[\log Q_w(n_{w,c} \mid n_{\mathrm{all},c}) - \log P(n_{w,c} \mid n_{\mathrm{all},c}, \hat{p}_w)\right], \tag{1}$$

where $P_w := P(n_w \mid n_{\mathrm{all}}, \hat{p}_w)$, and $Q_w(n_w \mid n_{\mathrm{all}})$ denotes the empirical distribution of $n_w$ for given $n_{\mathrm{all}}$ over the set of grid cells $\Gamma$.

In Fig SA1A, we present the estimated relative entropy for each toponym and each noun. As expected, toponyms denoting cities (such as "Nagoya") and some submunicipal districts (such as "Shinjuku"), as well as place nouns (such as "park"), are characterized by relatively larger values of relative entropy than those for common nouns without a place connotations (e.g., "wallet"), implying that the empirical distribution is highly dissimilar from the location-independent binomial model. However, the relative entropies for toponyms that represent regions (e.g., "Shikoku"), prefectures (e.g., "Ishikawa"), and some other submunicipal districts (e.g., "Setagaya") are as small as those for common nouns. This seems inconsistent with what we expect from S3 Fig in Supporting Information, in which the difference between the data and the model is clearly visible for all domestic toponyms. This is presumably because the data contain more grid cells without any toponym occurrence, i.e., $n_w = 0$, than expected by the model. Such cells are prevalent even in cells with large $n_{\mathrm{all}}$, in which the model predicts a low probability for $n_w = 0$. The increase in relative entropy due to the presence of such zero-occurrence grid cells will be more pronounced for words with a large $\hat{p}_w$, or,

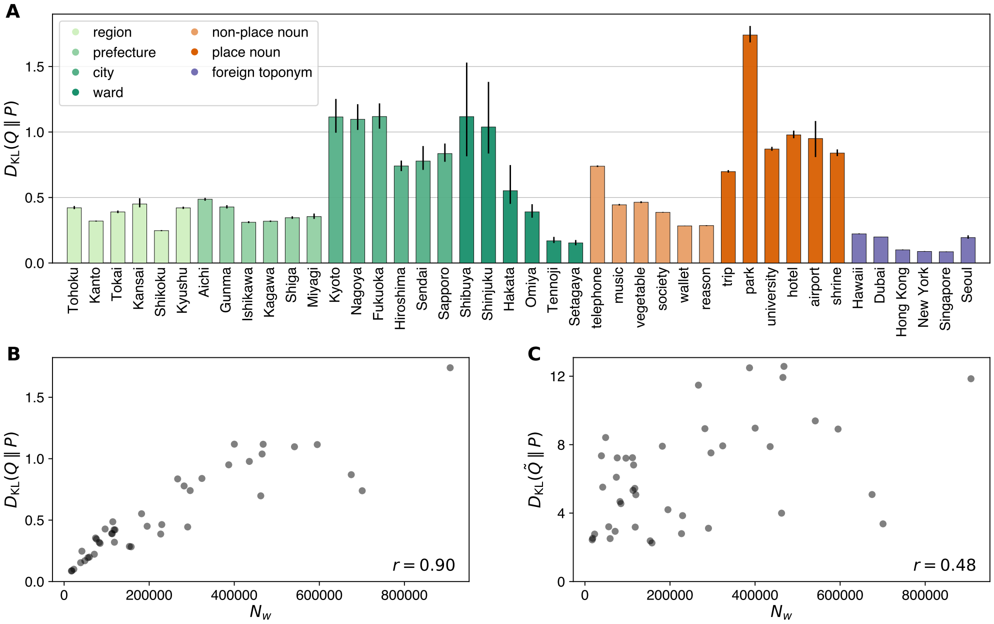


**Fig SA1. Dissimilarity of empirical data from the location-independent model, evaluated by relative entropy. (A)** Relative entropy $D_{\mathrm{KL}}(Q_w \parallel P_w)$ for each toponym $w$ and noun studied in this work. For each word, 20000 grid cells are randomly sampled 50 times to compute the mean and 95% confidence intervals, indicated by error bars. **(B, C)** Relative entropy $D_{\mathrm{KL}}(Q_w \parallel P_w)$ and the modified version $D_{\mathrm{KL}}(\tilde{Q}_w \parallel P_w)$, plotted against the total number of posts $N_w$ containing word $w$.

equivalently, a large number of posts $N_w$. This hypothesis is supported by the strong positive correlation between $N_w$ and $D_{\mathrm{KL}}(Q_w \parallel P_w)$, as shown in Fig SA1B. Simply put, a large value of relative entropy may just be an artifact of the popularity of the word.

To discount the effect of inflated zero occurrences, we only sample grid cells with non-zero occurrences for computing the empirical distribution and relative entropy, i.e., $\Gamma \subseteq \{c \mid n_{w,c} > 0\}$. Let $\tilde{Q}$ denote the empirical distribution thus obtained. As shown in Fig 5 in the main text, this approach clearly discriminates the domestic toponyms and place nouns from nonspatial nouns and foreign toponyms. The correlation between $N_w$ and $D_{\mathrm{KL}}(\tilde{Q}_w \parallel P_w)$ is much weaker (Fig SA1C), further corroborating the hypothesis that the value of $D_{\mathrm{KL}}(Q_w \parallel P_w)$ is dominated by the contribution from zero-occurrence grid cells.

# 2 Data preprocessing

In this section, we provide the details of the data preprocessing procedures.

## Selection of posts based on source applications

As described in the main text, we limit the sample to posts sent from one of the official or general-use third-party mobile applications. Specifically, posts that have ‘source’ metadata as one of the following applications are included: ‘Twitter for iPhone’, ‘Twitter for Android’, ‘Instagram’, ‘Twitter for iPad’, ‘Twitter for Android Tablets’, ‘Path’, ‘Mobile Web’, ‘Mobile Web (M5)’, ‘Path 2.0’, ‘ついっぷる for iPhone’, ‘Photos on iOS’, ‘Twitter for Windows Phone’, ‘ついっぷる Pro for iPhone’, ‘Twitter for

BlackBerry®', 'Camera on iOS', 'ついっぷる for Android', 'ついっぷる Pro for Android', 'ついっぷる for Android org', and 'ついっぷる Pro for Android org'. ついっぷる (Twipple) was a Twitter client provided by one of the major Japanese internet service providers, BIGLOBE, until it was discontinued in 2017.

## Unnatural geotag distribution

During the data cleaning process, we noticed that the geotags attached to some posts had an unnatural geographic distribution, likely due to manipulation. Figure SA2 shows the geographic distribution of posts containing different numbers of mentions (references to other accounts). There are several rectangular blocks clearly visible in the distributions of posts containing mentions of eight other accounts and nine or more accounts. These blocks are unlikely to be the result of organic distributions for two reasons: (1) some of them extend over sea areas, and (2) such patterns are absent in posts with fewer than seven mentions.

Figure SA3 provides further evidence of manipulation. When posts are categorized by their mention count, the number of posts in each category decreases monotonically with the number of mentions. However, the number of unique grid cells where posts in each category are distributed shows an increase at a mention count of seven. Although the geographic distribution of posts with seven mentions does not exhibit visually clear symptoms of manipulation on the map (Fig SA2), we opted to exclude all posts with seven or more mentions from the dataset as a conservative choice.

## Toponyms that are substrings of other toponyms

To exclude references to other toponyms from each toponym subsample, we identify the names of regions, prefectures, cities with a population larger than 50 000, and wards that contain each of the 24 toponyms we study in this work as a substring as follows:

- 関東 (Kanto) is a substring of 北関東 (Kitakanto) and 南関東 (Minamikanto).
- 四国 (Shikoku) is a substring of 四国中央 (Shikokuchuo).
- 九州 (Kyushu) is a substring of 北九州 (Kitakyushu).
- 宮城 (Miyagi) is a substring of 宮城野 (Miyagino).
- 京都 (Kyoto) is a substring of 東京都 (Tokyo-to, Tokyo Metropolis).
- 名古屋 (Nagoya) is a substring of 北名古屋 (Kitanagoya).
- 福岡 (Fukuoka) is a substring of 上福岡 (Kamifukuoka).
- 広島 (Hiroshima) is a substring of 北広島 (Kitahiroshima) and 東広島 (Higashihiroshima).

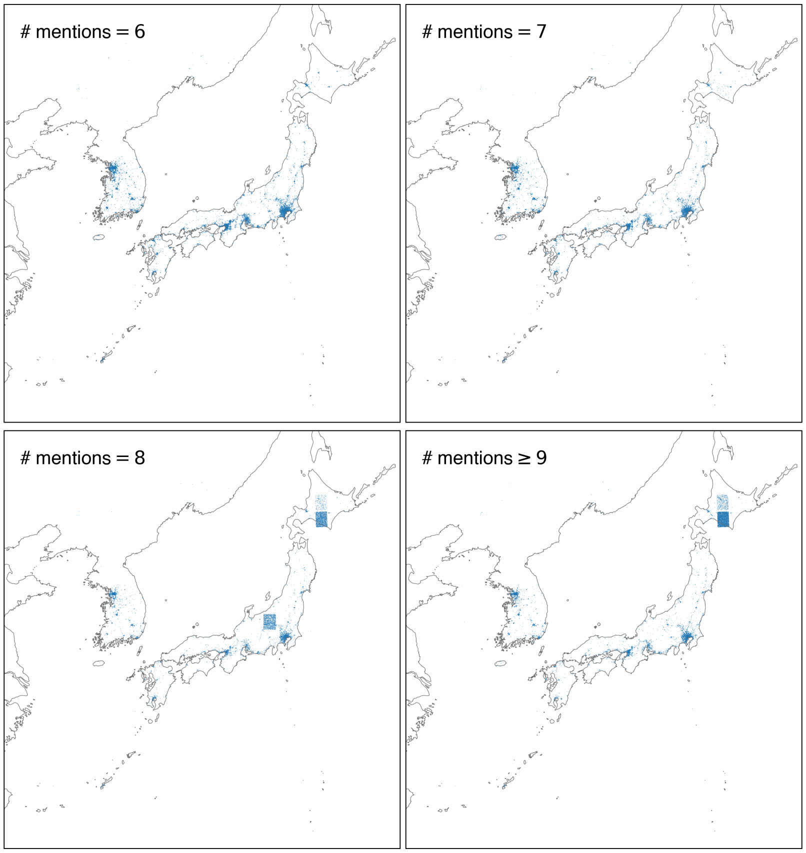


**Fig SA2. Spatial distributions of posts containing a specific number of mentions.** Grid cells to which a non-zero number of posts are tagged are colored in blue. Maps made with Natural Earth (`https://www.naturalearthdata.com/`).

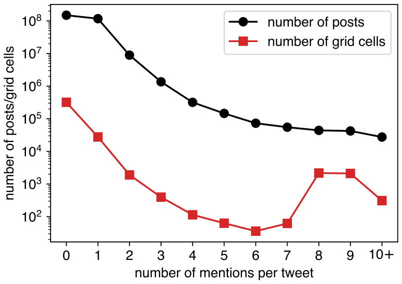


**Fig SA3. Number of posts containing a specific number of mentions, and the number of grid cells to which they are tagged.**
