# Supplementary figures and images for: Geospatial analysis of toponyms in geotagged social media posts
Source: PLoS One. 2025 Jun 5;20(6):e0325022. doi: 10.1371/journal.pone.0325022 (PMC12140283; doi:10.1371/journal.pone.0325022)

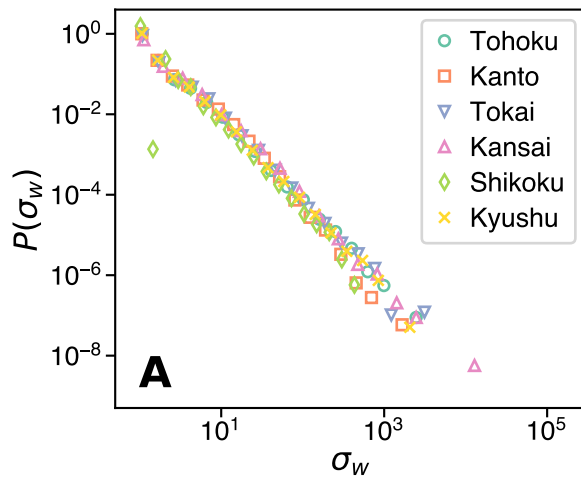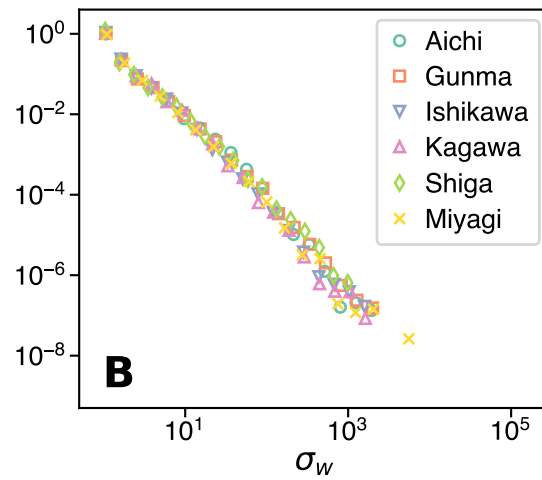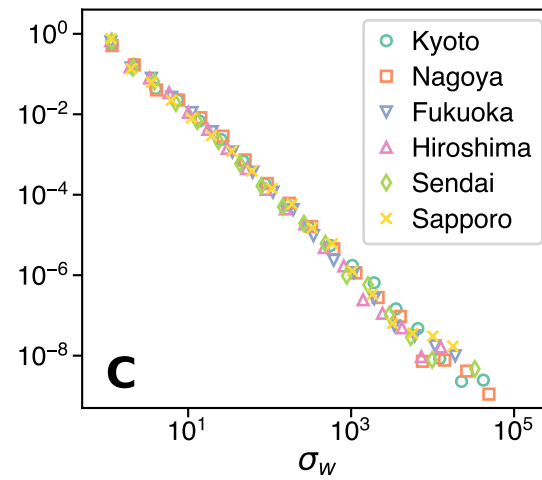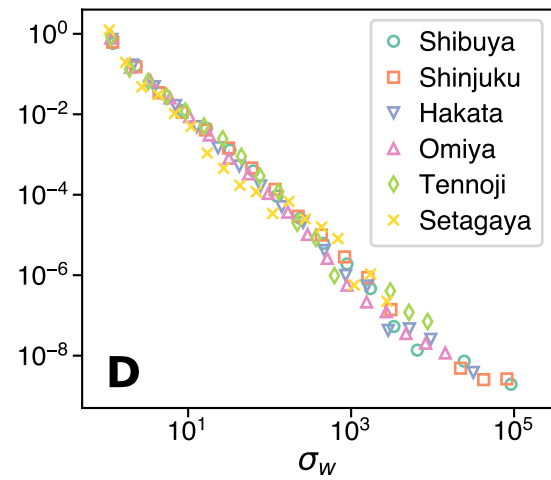

Supplement: S1 Fig — (PDF) [file pone.0325022.s002.pdf]

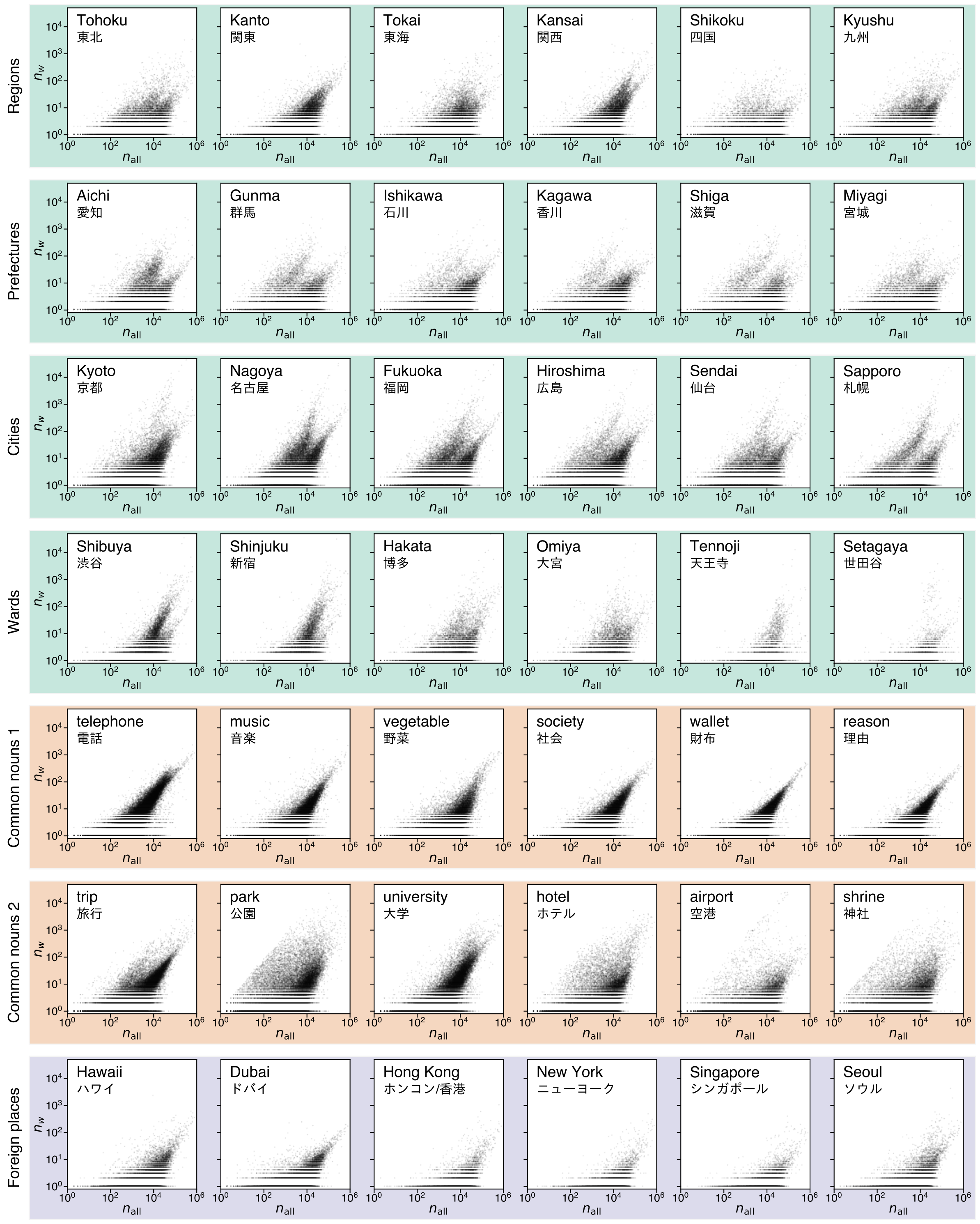

Supplement: S2 Fig — (PDF) [file pone.0325022.s003.pdf]

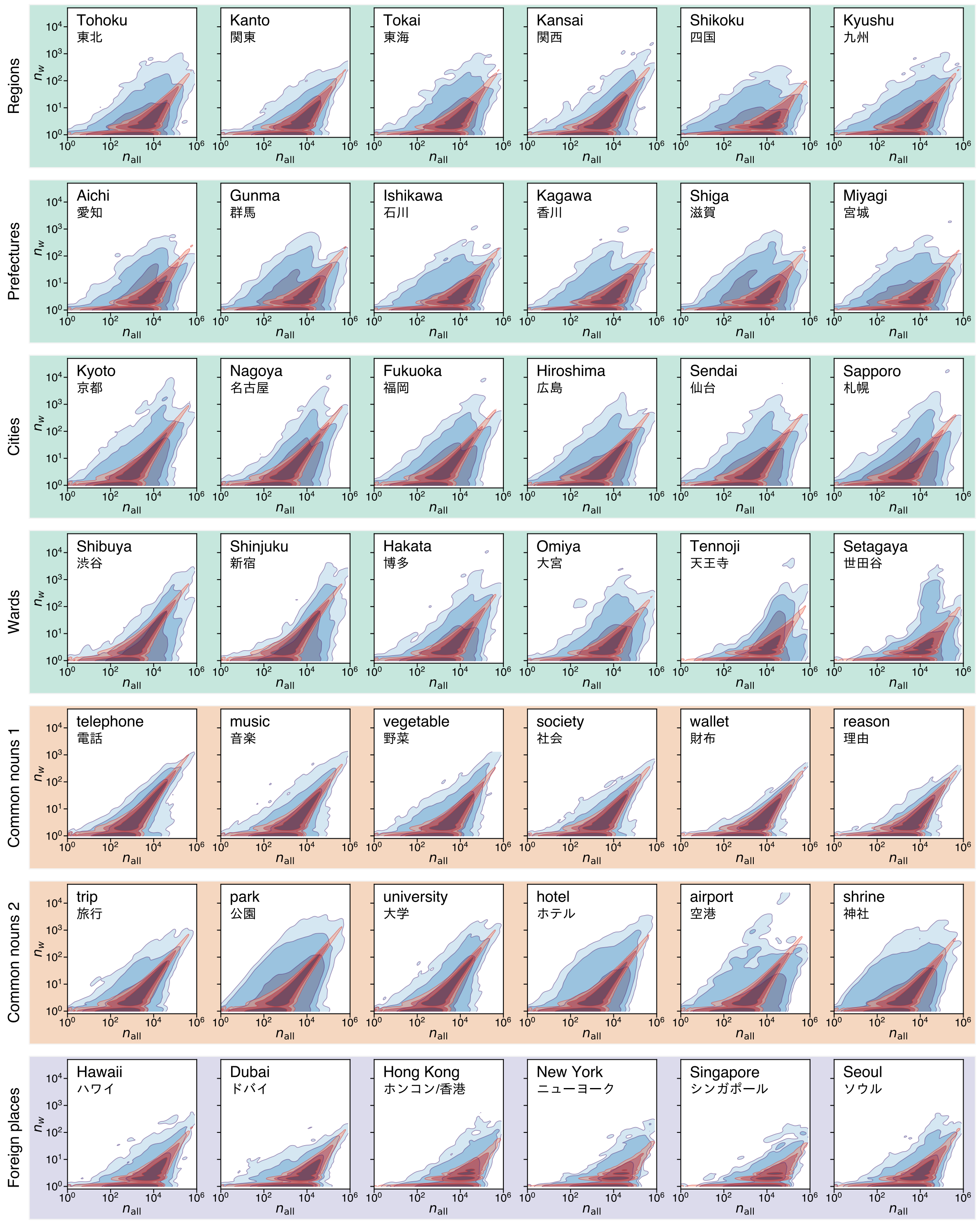

Supplement: S3 Fig — Each set of contours represents the kernel density plot of nw versus nall of empirical data (blue) and the location-independent model (red). (PDF) [file pone.0325022.s004.pdf]

Regions

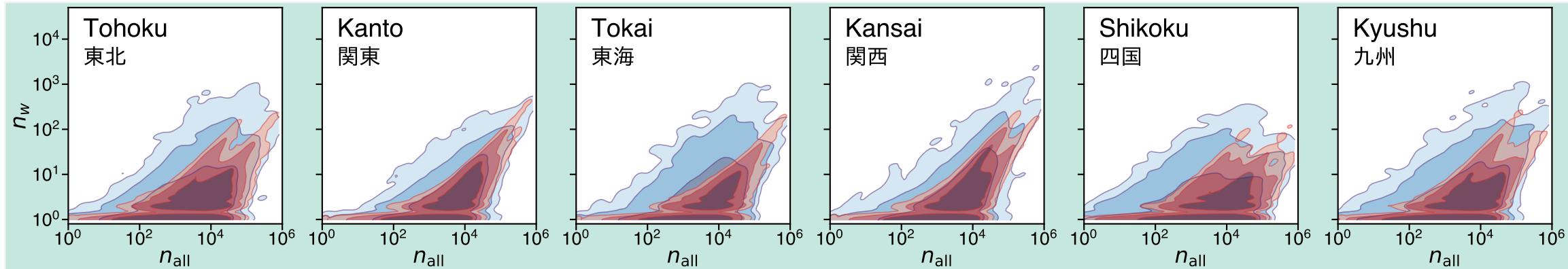

Prefectures

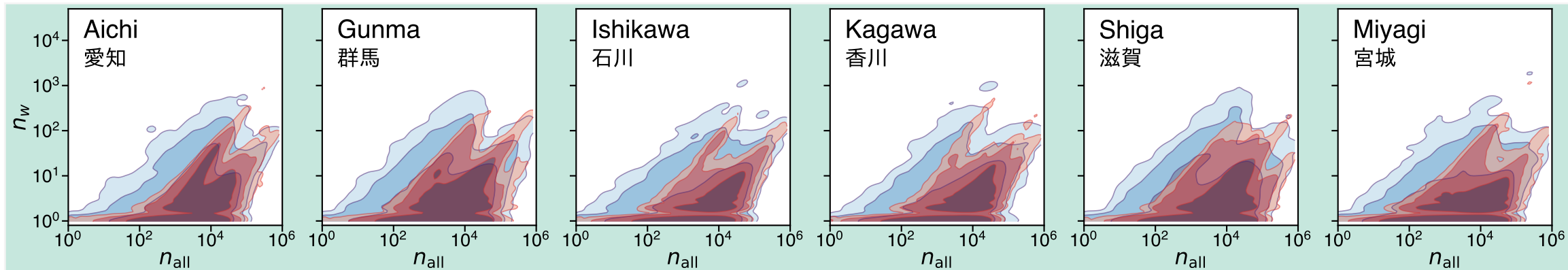

Cities

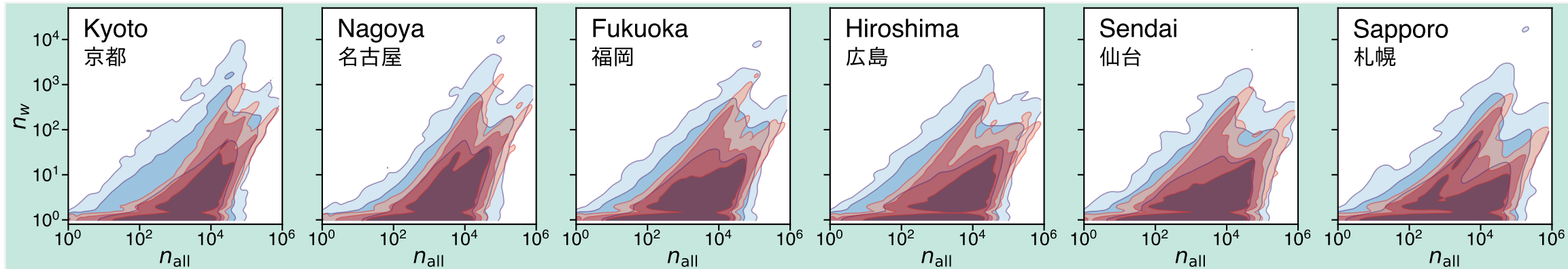

Wards

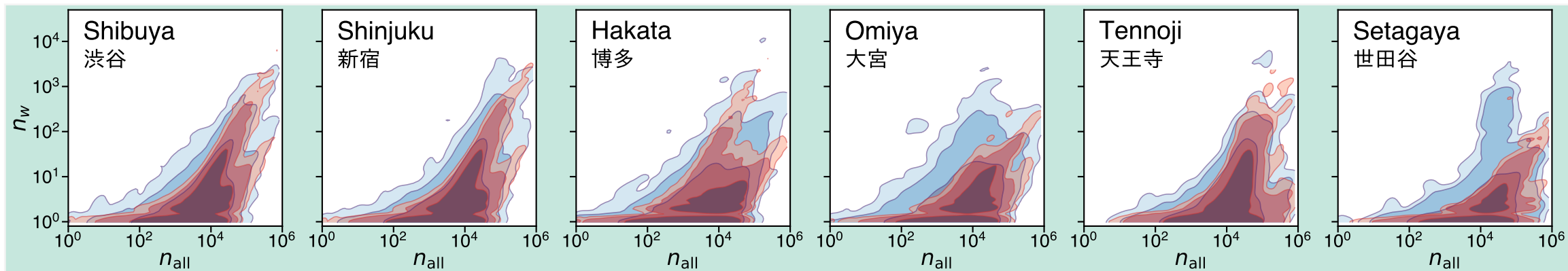

Supplement: S4 Fig — Each set of contours represents the kernel density plot of nw versus nall of empirical data (blue) and the core-periphery model (red). (PDF) [file pone.0325022.s005.pdf]
